# 1-Ferrocenyl-3-(4-methyl­anilino)propan-1-one

**DOI:** 10.1107/S1600536812003509

**Published:** 2012-01-31

**Authors:** Zorica Leka, Sladjana B. Novaković, Dragana Stevanović, Goran A. Bogdanović, Rastko D. Vukićević

**Affiliations:** a Faculty of Metallurgy and Technology, University of Montenegro, Cetinjski put bb, 81000 Podgorica, Montenegro; b ’Vinča’ Institute of Nuclear Sciences, Laboratory of Theoretical Physics and Condensed Matter Physics, PO Box 522, 11001 Belgrade, Serbia; c Department of Chemistry, Faculty of Science, University of Kragujevac, R. Domanovića 12, 34000 Kragujevac, Serbia

## Abstract

In the title ferrocene derivative, [Fe(C_5_H_5_)(C_15_H_16_NO)], the dihedral angle between the best planes of the benzene and the substituted cyclo­penta­dienyl ring is 83.4 (1)°. The presence of a methyl substituent in the *para* position of the aniline group does not alter the crystal packing compared to that of 3-anilino-1-ferrocenylpropan-1-one [Leka *et al.* (2012 ▶). *Acta Cryst.* E**68**, m229]. The molecules are connected into centro­symmetric dimers *via* N—H⋯O hydrogen bonds. In addition, C—H⋯O and C—H⋯N contacts stabilize the crystal packing.

## Related literature

For the physico-chemical properties of ferrocene-based compounds, see: Togni & Hayashi (1995 ▶). For related crystal structures and details of the synthesis, see: Damljanović *et al.* (2011 ▶); Stevanović *et al.* (2012 ▶); Leka *et al.* (2012*a* ▶,*b* ▶).
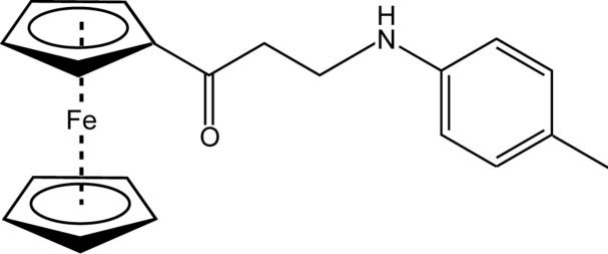


## Experimental

### 

#### Crystal data

[Fe(C_5_H_5_)(C_15_H_16_NO)]
*M* *_r_* = 347.23
Triclinic, 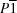

*a* = 7.553 (2) Å
*b* = 9.778 (3) Å
*c* = 13.640 (4) Å
α = 86.83 (2)°
β = 74.62 (3)°
γ = 67.71 (3)°
*V* = 897.6 (5) Å^3^
*Z* = 2
Mo *K*α radiation
μ = 0.84 mm^−1^
*T* = 293 K
0.22 × 0.18 × 0.16 mm

#### Data collection

Enraf–Nonius CAD-4 diffractometer
3804 measured reflections
3519 independent reflections
2829 reflections with *I* > 2σ(*I*)
*R*_int_ = 0.016
3 standard reflections every 60 min intensity decay: none

#### Refinement

*R*[*F* ^2^ > 2σ(*F* ^2^)] = 0.041
*wR*(*F* ^2^) = 0.115
*S* = 1.05
3519 reflections
213 parameters
H atoms treated by a mixture of independent and constrained refinement
Δρ_max_ = 0.41 e Å^−3^
Δρ_min_ = −0.41 e Å^−3^

### 

Data collection: *CAD-4 Software* (Enraf–Nonius, 1989 ▶); cell refinement: *CAD-4 Software*; data reduction: *CAD-4 Software*; program(s) used to solve structure: *SHELXS97* (Sheldrick, 2008 ▶); program(s) used to refine structure: *SHELXL97* (Sheldrick, 2008 ▶); molecular graphics: *ORTEP-3* (Farrugia, 1997 ▶) and *POV-RAY* (Persistence of Vision, 2004 ▶); software used to prepare material for publication: *WinGX* (Farrugia, 1999 ▶), *PLATON* (Spek, 2009 ▶) and *PARST* (Nardelli, 1995 ▶).

## Supplementary Material

Crystal structure: contains datablock(s) I, global. DOI: 10.1107/S1600536812003509/bt5791sup1.cif

Structure factors: contains datablock(s) I. DOI: 10.1107/S1600536812003509/bt5791Isup2.hkl

Additional supplementary materials:  crystallographic information; 3D view; checkCIF report

## Figures and Tables

**Table 1:** Hydrogen-bond geometry (Å, °)

*D*—H⋯*A*	*D*—H	H⋯*A*	*D*⋯*A*	*D*—H⋯*A*
N1—H1*N*⋯O1^i^	0.82 (3)	2.31 (4)	3.102 (4)	161 (3)
C19—H19⋯O1^i^	0.93	2.69	3.455 (4)	140
C4—H4⋯N1^ii^	0.93	2.64	3.451 (4)	147

Symmetry codes: (i) 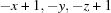; (ii) 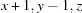.

**Fig. 1.**
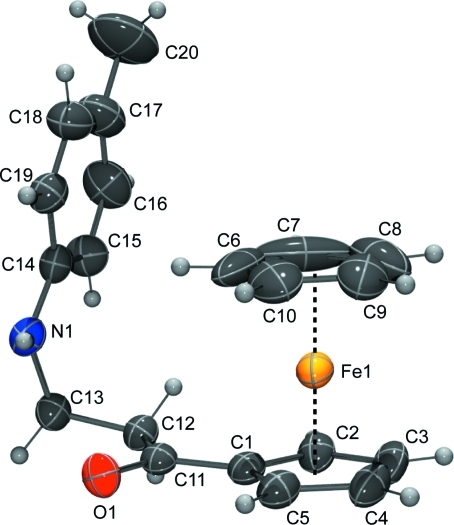
The molecular structure of the title compound, with atom labels and 40% probability displacement ellipsoids for non-H atoms.

**Fig. 2.**
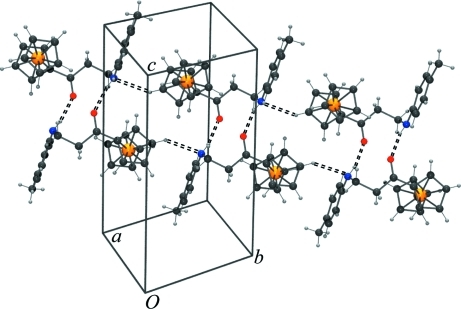
Part of the crystal packing showing the interconnection of dimers into a chain.

## supplementary crystallographic information

### Comment

The present report forms a part of our wider research of the structural properties of Mannich bases. The title compound (Figure 1) crystallizes in the same space group, *i.e. P*1, as the derivative containing an unsubstitued phenylamino moiety and exhibits very similar unit-cell parameters. The orientation of the cyclopentadienyl (Cp) rings slightly deviates from the eclipsed conformation as defined by the smallest torsion angle C—C*g*1—C*g*2—C of 4.9° (*Cg*1 and *Cg*2 are centroids of the corresponding Cp rings). The distances of Fe to C*g*1 and C*g*2 are 1.65 and 1.66°, respectively. The Cp rings are practically coplanar with the dihedral angle of 0.5 (2)°. The torsion angle O1—C11—C1—C5 which relates Cp1 ring with the carbonyl group is here equal to -6.2 (3)°, showing the expected co-planarity. Although C1—C11—C12—C13—N1 fragment consists of single bonds which allows for free rotation, the molecule adopts a conformation very similar to the previously reported derivatives (Damljanović *et al.*, 2011; Stevanović *et al.*, 2012). The bent conformation of the molecule is indicated by the C11—C12—C13— N1 torsion angle [(70.6 (3)°] which is slightly smaller than in the case of the molecule containing unmodified, phenylamino moiety. Regardless the fact that molecules of the present Mannich base contain an additional methyl subsistent in the *para* position of the phenylamino moiety their crystal packing arrangement (Figure 2) is closely similar to the previously reported 1-ferrocenyl-3-(phenylamino)propan-1-one (Leka *et al*., 2012*a*). As previously observed, the N—H···O bonded dimers represent the main structural feature of these Mannich basis. The methyl group does not take a part in the intermolecular interactions.

### Experimental

The compound was obtained in the reaction of aza-Michael addition of corresponding arylamine to acryloylferrocene. The reaction was performed by microwave (MW) irradiation (500 W/5 min) of a mixture of reactants and montmorillonite K-10, without a solvent as described by Damljanović *et al.* (2011).

### Refinement

H atoms bonded to C atoms were placed at geometrically calculated positions and refined using a riding model. C—H distances were fixed to 0.93, 0.97 and 0.96 Å from aromatic, methylene and methyl C atoms, respectively. The *U*_iso_(H) values were equal to 1.2 times *U*_eq_ of the corresponding aromatic C(*sp*^2^) and methylene C(*sp*^3^). The *U*_iso_(H) values of the H atoms attached to methyl C(*sp*^3^) were equal to 1.5 times *U*_eq_ of the parent atom. H atom attached to N atom was refined isotropically.

### Crystal data

**Table:**

[Fe(C_5_H_5_)(C_15_H_16_NO)]	*Z* = 2
*M**_r_* = 347.23	*F*(000) = 364
Triclinic, *P*1	*D*_x_ = 1.285 Mg m^−^^3^
Hall symbol: -P 1	Mo *K*α radiation, λ = 0.71073 Å
*a* = 7.553 (2) Å	Cell parameters from 25 reflections
*b* = 9.778 (3) Å	θ = 11.2–15.5°
*c* = 13.640 (4) Å	µ = 0.84 mm^−^^1^
α = 86.83 (2)°	*T* = 293 K
β = 74.62 (3)°	Prismatic, orange
γ = 67.71 (3)°	0.22 × 0.18 × 0.16 mm
*V* = 897.6 (5) Å^3^	

### Data collection

**Table:**

Enraf–Nonius CAD-4 diffractometer	*R*_int_ = 0.016
Radiation source: fine-focus sealed tube	θ_max_ = 26.0°, θ_min_ = 1.6°
graphite	*h* = 0→9
ω/2θ scans	*k* = −11→12
3804 measured reflections	*l* = −16→16
3519 independent reflections	3 standard reflections every 60 min
2829 reflections with *I* > 2σ(*I*)	intensity decay: none

### Refinement

**Table:**

Refinement on *F*^2^	Primary atom site location: structure-invariant direct methods
Least-squares matrix: full	Secondary atom site location: difference Fourier map
*R*[*F*^2^ > 2σ(*F*^2^)] = 0.041	Hydrogen site location: inferred from neighbouring sites
*wR*(*F*^2^) = 0.115	H atoms treated by a mixture of independent and constrained refinement
*S* = 1.05	*w* = 1/[σ^2^(*F*_o_^2^) + (0.065*P*)^2^ + 0.2205*P*] where *P* = (*F*_o_^2^ + 2*F*_c_^2^)/3
3519 reflections	(Δ/σ)_max_ < 0.001
213 parameters	Δρ_max_ = 0.41 e Å^−^^3^
0 restraints	Δρ_min_ = −0.40 e Å^−^^3^

### Fractional atomic coordinates and isotropic or equivalent isotropic displacement parameters (Å^2^)

**Table:**

	*x*	*y*	*z*	*U*_iso_*/*U*_eq_	
Fe1	0.82439 (5)	−0.29783 (4)	0.68484 (3)	0.04989 (14)	
O1	0.7296 (3)	−0.01261 (19)	0.48727 (13)	0.0550 (4)	
N1	0.3609 (3)	0.2268 (2)	0.63329 (17)	0.0487 (5)	
C1	0.9228 (3)	−0.1577 (2)	0.59236 (17)	0.0434 (5)	
C2	1.0021 (4)	−0.1826 (3)	0.6791 (2)	0.0522 (6)	
H2	0.9830	−0.1105	0.7269	0.063*	
C3	1.1144 (4)	−0.3355 (3)	0.6796 (2)	0.0626 (7)	
H3	1.1817	−0.3814	0.7278	0.075*	
C4	1.1072 (4)	−0.4068 (3)	0.5945 (2)	0.0619 (7)	
H4	1.1689	−0.5076	0.5771	0.074*	
C5	0.9901 (4)	−0.2987 (3)	0.5400 (2)	0.0525 (6)	
H5	0.9617	−0.3161	0.4807	0.063*	
C6	0.5242 (5)	−0.2197 (5)	0.7206 (5)	0.119 (2)	
H6	0.4437	−0.1340	0.6968	0.142*	
C7	0.5913 (10)	−0.2274 (8)	0.8092 (5)	0.154 (3)	
H7	0.5661	−0.1504	0.8545	0.185*	
C8	0.7072 (8)	−0.3819 (7)	0.8128 (3)	0.1120 (17)	
H8	0.7720	−0.4250	0.8625	0.134*	
C9	0.7059 (5)	−0.4533 (4)	0.7321 (3)	0.0885 (11)	
H9	0.7712	−0.5546	0.7169	0.106*	
C10	0.5963 (5)	−0.3570 (5)	0.6763 (3)	0.0930 (12)	
H10	0.5739	−0.3812	0.6169	0.112*	
C11	0.7851 (3)	−0.0189 (2)	0.56467 (17)	0.0416 (5)	
C12	0.7182 (3)	0.1184 (2)	0.63237 (18)	0.0452 (5)	
H12B	0.8298	0.1477	0.6260	0.054*	
H12A	0.6756	0.0955	0.7027	0.054*	
C13	0.5489 (4)	0.2475 (3)	0.6063 (2)	0.0497 (5)	
H13B	0.5316	0.3370	0.6416	0.060*	
H13A	0.5848	0.2613	0.5337	0.060*	
C14	0.2552 (4)	0.2300 (3)	0.73370 (19)	0.0466 (5)	
C15	0.2798 (5)	0.2977 (3)	0.8142 (2)	0.0643 (7)	
H15	0.3764	0.3382	0.8020	0.077*	
C16	0.1612 (6)	0.3046 (4)	0.9115 (2)	0.0861 (10)	
H16	0.1805	0.3503	0.9636	0.103*	
C17	0.0152 (6)	0.2468 (4)	0.9352 (3)	0.0878 (10)	
C18	−0.0069 (5)	0.1779 (4)	0.8545 (3)	0.0789 (9)	
H18	−0.1029	0.1367	0.8673	0.095*	
C19	0.1089 (4)	0.1693 (3)	0.7571 (2)	0.0575 (6)	
H19	0.0901	0.1223	0.7055	0.069*	
C20	−0.1148 (9)	0.2559 (7)	1.0428 (3)	0.154 (2)	
H20A	−0.0632	0.2899	1.0895	0.230*	
H20B	−0.1157	0.1597	1.0605	0.230*	
H20C	−0.2477	0.3237	1.0464	0.230*	
H1N	0.366 (4)	0.163 (3)	0.595 (2)	0.047 (7)*	

### Atomic displacement parameters (Å^2^)

**Table:**

	*U*^11^	*U*^22^	*U*^33^	*U*^12^	*U*^13^	*U*^23^
Fe1	0.0419 (2)	0.0508 (2)	0.0630 (2)	−0.02310 (15)	−0.01730 (16)	0.01293 (16)
O1	0.0657 (11)	0.0563 (10)	0.0499 (9)	−0.0244 (9)	−0.0245 (8)	0.0024 (8)
N1	0.0461 (11)	0.0494 (11)	0.0521 (12)	−0.0159 (9)	−0.0168 (9)	−0.0059 (9)
C1	0.0377 (11)	0.0467 (12)	0.0505 (12)	−0.0219 (9)	−0.0105 (9)	0.0024 (10)
C2	0.0483 (13)	0.0542 (14)	0.0653 (15)	−0.0255 (11)	−0.0246 (12)	0.0070 (12)
C3	0.0440 (13)	0.0614 (16)	0.090 (2)	−0.0211 (12)	−0.0318 (13)	0.0194 (14)
C4	0.0434 (13)	0.0440 (13)	0.093 (2)	−0.0129 (11)	−0.0158 (13)	0.0033 (13)
C5	0.0456 (13)	0.0493 (13)	0.0637 (15)	−0.0210 (11)	−0.0105 (11)	−0.0018 (11)
C6	0.0384 (16)	0.082 (3)	0.202 (6)	−0.0174 (17)	0.005 (2)	0.052 (3)
C7	0.138 (5)	0.176 (6)	0.143 (5)	−0.121 (5)	0.084 (4)	−0.089 (4)
C8	0.116 (3)	0.182 (5)	0.082 (3)	−0.103 (4)	−0.036 (2)	0.053 (3)
C9	0.074 (2)	0.081 (2)	0.127 (3)	−0.0502 (19)	−0.029 (2)	0.037 (2)
C10	0.067 (2)	0.133 (4)	0.110 (3)	−0.067 (2)	−0.034 (2)	0.036 (3)
C11	0.0382 (11)	0.0468 (12)	0.0458 (12)	−0.0232 (9)	−0.0104 (9)	0.0030 (9)
C12	0.0429 (12)	0.0456 (12)	0.0517 (13)	−0.0192 (10)	−0.0159 (10)	0.0001 (10)
C13	0.0518 (13)	0.0409 (12)	0.0584 (14)	−0.0187 (10)	−0.0163 (11)	0.0043 (10)
C14	0.0454 (12)	0.0420 (12)	0.0518 (13)	−0.0117 (10)	−0.0190 (11)	0.0020 (10)
C15	0.0695 (18)	0.0727 (18)	0.0577 (16)	−0.0324 (14)	−0.0173 (13)	−0.0090 (13)
C16	0.110 (3)	0.098 (3)	0.0526 (17)	−0.040 (2)	−0.0206 (18)	−0.0092 (16)
C17	0.094 (3)	0.092 (2)	0.0623 (19)	−0.033 (2)	−0.0009 (18)	0.0047 (17)
C18	0.070 (2)	0.084 (2)	0.082 (2)	−0.0381 (17)	−0.0089 (17)	0.0137 (17)
C19	0.0534 (14)	0.0577 (15)	0.0657 (16)	−0.0227 (12)	−0.0196 (12)	0.0005 (12)
C20	0.172 (6)	0.180 (6)	0.074 (3)	−0.069 (5)	0.028 (3)	−0.001 (3)

### Geometric parameters (Å, °)

**Table:**

Fe1—C7	2.019 (4)	C7—C8	1.436 (8)
Fe1—C8	2.020 (4)	C7—H7	0.9300
Fe1—C6	2.025 (3)	C8—C9	1.339 (6)
Fe1—C1	2.026 (2)	C8—H8	0.9300
Fe1—C9	2.036 (3)	C9—C10	1.347 (5)
Fe1—C5	2.037 (3)	C9—H9	0.9300
Fe1—C2	2.040 (2)	C10—H10	0.9300
Fe1—C10	2.046 (3)	C11—C12	1.512 (3)
Fe1—C4	2.059 (3)	C12—C13	1.524 (3)
Fe1—C3	2.061 (3)	C12—H12B	0.9700
O1—C11	1.225 (3)	C12—H12A	0.9700
N1—C14	1.386 (3)	C13—H13B	0.9700
N1—C13	1.458 (3)	C13—H13A	0.9700
N1—H1N	0.82 (3)	C14—C15	1.400 (4)
C1—C2	1.433 (3)	C14—C19	1.401 (4)
C1—C5	1.433 (3)	C15—C16	1.380 (5)
C1—C11	1.466 (3)	C15—H15	0.9300
C2—C3	1.413 (4)	C16—C17	1.379 (6)
C2—H2	0.9300	C16—H16	0.9300
C3—C4	1.410 (4)	C17—C18	1.400 (5)
C3—H3	0.9300	C17—C20	1.520 (5)
C4—C5	1.416 (4)	C18—C19	1.370 (4)
C4—H4	0.9300	C18—H18	0.9300
C5—H5	0.9300	C19—H19	0.9300
C6—C10	1.351 (7)	C20—H20A	0.9600
C6—C7	1.417 (8)	C20—H20B	0.9600
C6—H6	0.9300	C20—H20C	0.9600
			
C7—Fe1—C8	41.7 (2)	C1—C5—Fe1	68.92 (14)
C7—Fe1—C6	41.0 (2)	C4—C5—H5	126.0
C8—Fe1—C6	67.5 (2)	C1—C5—H5	126.0
C7—Fe1—C1	122.9 (2)	Fe1—C5—H5	126.1
C8—Fe1—C1	160.1 (2)	C10—C6—C7	108.9 (4)
C6—Fe1—C1	109.62 (13)	C10—C6—Fe1	71.4 (2)
C7—Fe1—C9	67.37 (19)	C7—C6—Fe1	69.3 (2)
C8—Fe1—C9	38.55 (19)	C10—C6—H6	125.5
C6—Fe1—C9	65.48 (16)	C7—C6—H6	125.5
C1—Fe1—C9	159.90 (15)	Fe1—C6—H6	125.3
C7—Fe1—C5	158.2 (3)	C6—C7—C8	103.8 (4)
C8—Fe1—C5	157.6 (2)	C6—C7—Fe1	69.7 (2)
C6—Fe1—C5	121.7 (2)	C8—C7—Fe1	69.2 (2)
C1—Fe1—C5	41.30 (10)	C6—C7—H7	128.1
C9—Fe1—C5	122.86 (15)	C8—C7—H7	128.1
C7—Fe1—C2	109.55 (16)	Fe1—C7—H7	124.7
C8—Fe1—C2	123.88 (17)	C9—C8—C7	108.2 (4)
C6—Fe1—C2	128.16 (18)	C9—C8—Fe1	71.4 (2)
C1—Fe1—C2	41.26 (10)	C7—C8—Fe1	69.2 (2)
C9—Fe1—C2	157.13 (14)	C9—C8—H8	125.9
C5—Fe1—C2	68.80 (11)	C7—C8—H8	125.9
C7—Fe1—C10	67.3 (2)	Fe1—C8—H8	125.1
C8—Fe1—C10	65.57 (17)	C8—C9—C10	110.1 (4)
C6—Fe1—C10	38.8 (2)	C8—C9—Fe1	70.1 (2)
C1—Fe1—C10	125.38 (13)	C10—C9—Fe1	71.1 (2)
C9—Fe1—C10	38.54 (15)	C8—C9—H9	125.0
C5—Fe1—C10	107.63 (15)	C10—C9—H9	125.0
C2—Fe1—C10	163.31 (14)	Fe1—C9—H9	125.4
C7—Fe1—C4	160.8 (3)	C9—C10—C6	109.0 (5)
C8—Fe1—C4	122.6 (2)	C9—C10—Fe1	70.33 (19)
C6—Fe1—C4	155.2 (2)	C6—C10—Fe1	69.8 (2)
C1—Fe1—C4	68.69 (10)	C9—C10—H10	125.5
C9—Fe1—C4	107.10 (14)	C6—C10—H10	125.5
C5—Fe1—C4	40.44 (11)	Fe1—C10—H10	125.9
C2—Fe1—C4	67.92 (11)	O1—C11—C1	121.2 (2)
C10—Fe1—C4	120.67 (17)	O1—C11—C12	120.6 (2)
C7—Fe1—C3	125.8 (3)	C1—C11—C12	118.20 (19)
C8—Fe1—C3	108.53 (16)	C11—C12—C13	112.77 (19)
C6—Fe1—C3	164.3 (2)	C11—C12—H12B	109.0
C1—Fe1—C3	68.62 (10)	C13—C12—H12B	109.0
C9—Fe1—C3	121.62 (14)	C11—C12—H12A	109.0
C5—Fe1—C3	67.99 (12)	C13—C12—H12A	109.0
C2—Fe1—C3	40.30 (11)	H12B—C12—H12A	107.8
C10—Fe1—C3	155.02 (16)	N1—C13—C12	113.48 (19)
C4—Fe1—C3	40.02 (12)	N1—C13—H13B	108.9
C14—N1—C13	121.9 (2)	C12—C13—H13B	108.9
C14—N1—H1N	116.2 (19)	N1—C13—H13A	108.9
C13—N1—H1N	109.9 (19)	C12—C13—H13A	108.9
C2—C1—C5	107.0 (2)	H13B—C13—H13A	107.7
C2—C1—C11	127.8 (2)	N1—C14—C15	123.3 (2)
C5—C1—C11	125.0 (2)	N1—C14—C19	119.4 (2)
C2—C1—Fe1	69.89 (13)	C15—C14—C19	117.2 (2)
C5—C1—Fe1	69.78 (14)	C16—C15—C14	120.3 (3)
C11—C1—Fe1	121.59 (15)	C16—C15—H15	119.9
C3—C2—C1	108.1 (2)	C14—C15—H15	119.9
C3—C2—Fe1	70.65 (14)	C17—C16—C15	123.0 (3)
C1—C2—Fe1	68.85 (13)	C17—C16—H16	118.5
C3—C2—H2	125.9	C15—C16—H16	118.5
C1—C2—H2	125.9	C16—C17—C18	116.4 (3)
Fe1—C2—H2	126.1	C16—C17—C20	122.3 (4)
C4—C3—C2	108.5 (2)	C18—C17—C20	121.3 (4)
C4—C3—Fe1	69.92 (15)	C19—C18—C17	121.9 (3)
C2—C3—Fe1	69.06 (14)	C19—C18—H18	119.1
C4—C3—H3	125.8	C17—C18—H18	119.1
C2—C3—H3	125.8	C18—C19—C14	121.3 (3)
Fe1—C3—H3	126.8	C18—C19—H19	119.4
C3—C4—C5	108.4 (2)	C14—C19—H19	119.4
C3—C4—Fe1	70.06 (16)	C17—C20—H20A	109.5
C5—C4—Fe1	68.96 (15)	C17—C20—H20B	109.5
C3—C4—H4	125.8	H20A—C20—H20B	109.5
C5—C4—H4	125.8	C17—C20—H20C	109.5
Fe1—C4—H4	126.7	H20A—C20—H20C	109.5
C4—C5—C1	108.0 (2)	H20B—C20—H20C	109.5
C4—C5—Fe1	70.60 (17)		
			
C7—Fe1—C1—C2	82.4 (3)	C2—Fe1—C6—C10	165.2 (2)
C8—Fe1—C1—C2	48.2 (5)	C4—Fe1—C6—C10	41.1 (5)
C6—Fe1—C1—C2	126.0 (3)	C3—Fe1—C6—C10	−156.8 (4)
C9—Fe1—C1—C2	−161.9 (4)	C8—Fe1—C6—C7	41.0 (3)
C5—Fe1—C1—C2	−117.8 (2)	C1—Fe1—C6—C7	−118.0 (3)
C10—Fe1—C1—C2	166.4 (2)	C9—Fe1—C6—C7	83.1 (3)
C4—Fe1—C1—C2	−80.38 (17)	C5—Fe1—C6—C7	−162.1 (3)
C3—Fe1—C1—C2	−37.27 (16)	C2—Fe1—C6—C7	−75.2 (3)
C7—Fe1—C1—C5	−159.8 (3)	C10—Fe1—C6—C7	119.5 (4)
C8—Fe1—C1—C5	166.0 (5)	C4—Fe1—C6—C7	160.6 (4)
C6—Fe1—C1—C5	−116.1 (3)	C3—Fe1—C6—C7	−37.3 (6)
C9—Fe1—C1—C5	−44.1 (4)	C10—C6—C7—C8	−0.5 (4)
C2—Fe1—C1—C5	117.8 (2)	Fe1—C6—C7—C8	−61.2 (3)
C10—Fe1—C1—C5	−75.8 (2)	C10—C6—C7—Fe1	60.7 (3)
C4—Fe1—C1—C5	37.45 (16)	C8—Fe1—C7—C6	−114.4 (4)
C3—Fe1—C1—C5	80.56 (17)	C1—Fe1—C7—C6	82.3 (3)
C7—Fe1—C1—C11	−40.4 (4)	C9—Fe1—C7—C6	−78.1 (3)
C8—Fe1—C1—C11	−74.6 (5)	C5—Fe1—C7—C6	44.6 (5)
C6—Fe1—C1—C11	3.2 (3)	C2—Fe1—C7—C6	126.2 (3)
C9—Fe1—C1—C11	75.3 (4)	C10—Fe1—C7—C6	−36.2 (3)
C5—Fe1—C1—C11	119.4 (2)	C4—Fe1—C7—C6	−154.9 (4)
C2—Fe1—C1—C11	−122.8 (2)	C3—Fe1—C7—C6	168.3 (2)
C10—Fe1—C1—C11	43.6 (3)	C6—Fe1—C7—C8	114.4 (4)
C4—Fe1—C1—C11	156.8 (2)	C1—Fe1—C7—C8	−163.3 (2)
C3—Fe1—C1—C11	−160.1 (2)	C9—Fe1—C7—C8	36.3 (3)
C5—C1—C2—C3	−0.2 (3)	C5—Fe1—C7—C8	159.0 (3)
C11—C1—C2—C3	174.9 (2)	C2—Fe1—C7—C8	−119.4 (3)
Fe1—C1—C2—C3	59.96 (18)	C10—Fe1—C7—C8	78.2 (3)
C5—C1—C2—Fe1	−60.20 (16)	C4—Fe1—C7—C8	−40.5 (6)
C11—C1—C2—Fe1	115.0 (2)	C3—Fe1—C7—C8	−77.3 (3)
C7—Fe1—C2—C3	122.7 (4)	C6—C7—C8—C9	0.6 (4)
C8—Fe1—C2—C3	78.5 (3)	Fe1—C7—C8—C9	−61.0 (3)
C6—Fe1—C2—C3	165.0 (3)	C6—C7—C8—Fe1	61.6 (3)
C1—Fe1—C2—C3	−119.3 (2)	C7—Fe1—C8—C9	118.8 (4)
C9—Fe1—C2—C3	44.8 (4)	C6—Fe1—C8—C9	78.4 (3)
C5—Fe1—C2—C3	−80.56 (19)	C1—Fe1—C8—C9	163.9 (3)
C10—Fe1—C2—C3	−161.2 (5)	C5—Fe1—C8—C9	−40.7 (5)
C4—Fe1—C2—C3	−36.92 (18)	C2—Fe1—C8—C9	−159.8 (2)
C7—Fe1—C2—C1	−118.0 (3)	C10—Fe1—C8—C9	36.1 (3)
C8—Fe1—C2—C1	−162.2 (2)	C4—Fe1—C8—C9	−75.9 (3)
C6—Fe1—C2—C1	−75.6 (3)	C3—Fe1—C8—C9	−117.8 (3)
C9—Fe1—C2—C1	164.1 (4)	C6—Fe1—C8—C7	−40.3 (3)
C5—Fe1—C2—C1	38.75 (14)	C1—Fe1—C8—C7	45.2 (6)
C10—Fe1—C2—C1	−41.9 (5)	C9—Fe1—C8—C7	−118.8 (4)
C4—Fe1—C2—C1	82.40 (16)	C5—Fe1—C8—C7	−159.5 (4)
C3—Fe1—C2—C1	119.3 (2)	C2—Fe1—C8—C7	81.5 (4)
C1—C2—C3—C4	0.1 (3)	C10—Fe1—C8—C7	−82.7 (4)
Fe1—C2—C3—C4	58.98 (19)	C4—Fe1—C8—C7	165.3 (3)
C1—C2—C3—Fe1	−58.84 (17)	C3—Fe1—C8—C7	123.4 (3)
C7—Fe1—C3—C4	162.2 (3)	C7—C8—C9—C10	−0.5 (4)
C8—Fe1—C3—C4	119.0 (3)	Fe1—C8—C9—C10	−60.1 (3)
C6—Fe1—C3—C4	−168.5 (5)	C7—C8—C9—Fe1	59.6 (3)
C1—Fe1—C3—C4	−81.91 (17)	C7—Fe1—C9—C8	−39.1 (4)
C9—Fe1—C3—C4	78.7 (2)	C6—Fe1—C9—C8	−84.1 (4)
C5—Fe1—C3—C4	−37.31 (16)	C1—Fe1—C9—C8	−164.1 (4)
C2—Fe1—C3—C4	−120.1 (2)	C5—Fe1—C9—C8	162.7 (3)
C10—Fe1—C3—C4	47.3 (4)	C2—Fe1—C9—C8	47.7 (5)
C7—Fe1—C3—C2	−77.8 (3)	C10—Fe1—C9—C8	−120.7 (4)
C8—Fe1—C3—C2	−120.9 (3)	C4—Fe1—C9—C8	121.3 (3)
C6—Fe1—C3—C2	−48.4 (6)	C3—Fe1—C9—C8	80.0 (3)
C1—Fe1—C3—C2	38.14 (16)	C7—Fe1—C9—C10	81.5 (4)
C9—Fe1—C3—C2	−161.3 (2)	C8—Fe1—C9—C10	120.7 (4)
C5—Fe1—C3—C2	82.74 (18)	C6—Fe1—C9—C10	36.6 (3)
C10—Fe1—C3—C2	167.4 (3)	C1—Fe1—C9—C10	−43.4 (5)
C4—Fe1—C3—C2	120.1 (2)	C5—Fe1—C9—C10	−76.6 (3)
C2—C3—C4—C5	0.0 (3)	C2—Fe1—C9—C10	168.3 (3)
Fe1—C3—C4—C5	58.46 (18)	C4—Fe1—C9—C10	−118.0 (3)
C2—C3—C4—Fe1	−58.45 (19)	C3—Fe1—C9—C10	−159.3 (3)
C7—Fe1—C4—C3	−49.0 (5)	C8—C9—C10—C6	0.1 (4)
C8—Fe1—C4—C3	−79.9 (2)	Fe1—C9—C10—C6	−59.3 (2)
C6—Fe1—C4—C3	172.6 (3)	C8—C9—C10—Fe1	59.4 (3)
C1—Fe1—C4—C3	81.73 (16)	C7—C6—C10—C9	0.3 (4)
C9—Fe1—C4—C3	−119.1 (2)	Fe1—C6—C10—C9	59.6 (3)
C5—Fe1—C4—C3	119.9 (2)	C7—C6—C10—Fe1	−59.3 (3)
C2—Fe1—C4—C3	37.16 (16)	C7—Fe1—C10—C9	−81.7 (4)
C10—Fe1—C4—C3	−158.85 (19)	C8—Fe1—C10—C9	−36.1 (3)
C7—Fe1—C4—C5	−169.0 (4)	C6—Fe1—C10—C9	−119.9 (4)
C8—Fe1—C4—C5	160.2 (2)	C1—Fe1—C10—C9	163.2 (2)
C6—Fe1—C4—C5	52.6 (4)	C5—Fe1—C10—C9	121.0 (3)
C1—Fe1—C4—C5	−38.22 (15)	C2—Fe1—C10—C9	−164.1 (4)
C9—Fe1—C4—C5	120.94 (19)	C4—Fe1—C10—C9	78.7 (3)
C2—Fe1—C4—C5	−82.78 (16)	C3—Fe1—C10—C9	45.4 (5)
C10—Fe1—C4—C5	81.2 (2)	C7—Fe1—C10—C6	38.3 (3)
C3—Fe1—C4—C5	−119.9 (2)	C8—Fe1—C10—C6	83.9 (4)
C3—C4—C5—C1	−0.2 (3)	C1—Fe1—C10—C6	−76.9 (3)
Fe1—C4—C5—C1	58.98 (17)	C9—Fe1—C10—C6	119.9 (4)
C3—C4—C5—Fe1	−59.1 (2)	C5—Fe1—C10—C6	−119.1 (3)
C2—C1—C5—C4	0.2 (3)	C2—Fe1—C10—C6	−44.2 (6)
C11—C1—C5—C4	−175.1 (2)	C4—Fe1—C10—C6	−161.3 (3)
Fe1—C1—C5—C4	−60.03 (18)	C3—Fe1—C10—C6	165.3 (4)
C2—C1—C5—Fe1	60.27 (16)	C2—C1—C11—O1	179.4 (2)
C11—C1—C5—Fe1	−115.1 (2)	C5—C1—C11—O1	−6.2 (3)
C7—Fe1—C5—C4	170.3 (4)	Fe1—C1—C11—O1	−92.5 (2)
C8—Fe1—C5—C4	−48.4 (4)	C2—C1—C11—C12	1.5 (3)
C6—Fe1—C5—C4	−156.9 (2)	C5—C1—C11—C12	175.9 (2)
C1—Fe1—C5—C4	119.1 (2)	Fe1—C1—C11—C12	89.6 (2)
C9—Fe1—C5—C4	−77.4 (2)	O1—C11—C12—C13	12.3 (3)
C2—Fe1—C5—C4	80.42 (17)	C1—C11—C12—C13	−169.82 (19)
C10—Fe1—C5—C4	−116.9 (2)	C14—N1—C13—C12	69.9 (3)
C3—Fe1—C5—C4	36.94 (16)	C11—C12—C13—N1	70.6 (3)
C7—Fe1—C5—C1	51.1 (5)	C13—N1—C14—C15	21.2 (4)
C8—Fe1—C5—C1	−167.6 (4)	C13—N1—C14—C19	−162.0 (2)
C6—Fe1—C5—C1	83.9 (2)	N1—C14—C15—C16	176.1 (3)
C9—Fe1—C5—C1	163.46 (17)	C19—C14—C15—C16	−0.8 (4)
C2—Fe1—C5—C1	−38.73 (14)	C14—C15—C16—C17	0.0 (6)
C10—Fe1—C5—C1	123.97 (19)	C15—C16—C17—C18	0.6 (6)
C4—Fe1—C5—C1	−119.1 (2)	C15—C16—C17—C20	−179.8 (4)
C3—Fe1—C5—C1	−82.20 (16)	C16—C17—C18—C19	−0.6 (6)
C7—Fe1—C6—C10	−119.5 (4)	C20—C17—C18—C19	179.8 (4)
C8—Fe1—C6—C10	−78.6 (3)	C17—C18—C19—C14	−0.2 (5)
C1—Fe1—C6—C10	122.5 (3)	N1—C14—C19—C18	−176.1 (3)
C9—Fe1—C6—C10	−36.4 (3)	C15—C14—C19—C18	0.9 (4)
C5—Fe1—C6—C10	78.4 (3)		

### Hydrogen-bond geometry (Å, °)

**Table:**

*D*—H···*A*	*D*—H	H···*A*	*D*···*A*	*D*—H···*A*
N1—H1N···O1^i^	0.82 (3)	2.31 (4)	3.102 (4)	161 (3)
C19—H19···O1^i^	0.93	2.69	3.455 (4)	140
C4—H4···N1^ii^	0.93	2.64	3.451 (4)	147

Symmetry codes: (i) −*x*+1, −*y*, −*z*+1; (ii) *x*+1, *y*−1, *z*.
